# Genetic and Epigenetic Sexual Dimorphism of Brain Cells during Aging

**DOI:** 10.3390/brainsci13020195

**Published:** 2023-01-24

**Authors:** Olesya Shirokova, Olga Zaborskaya, Pavel Pchelin, Elizaveta Kozliaeva, Vladimir Pershin, Irina Mukhina

**Affiliations:** 1 Institute of Fundamental Medicine, Privolzhsky Research Medical University, 10/1 Minin and Pozharsky Square, Nizhny Novgorod 603950, Russia; 2 Institute of Biology and Biomedicine, Lobachevsky State University, 23 Gagarin Avenue, Nizhny Novgorod 603002, Russia

**Keywords:** brain, aging, genetic, epigenetic, sexual dimorphism

## Abstract

In recent years, much of the attention paid to theoretical and applied biomedicine, as well as neurobiology, has been drawn to various aspects of sexual dimorphism due to the differences that male and female brain cells demonstrate during aging: (a) a dimorphic pattern of response to therapy for neurodegenerative disorders, (b) different age of onset and different degrees of the prevalence of such disorders, and (c) differences in their symptomatic manifestations in men and women. The purpose of this review is to outline the genetic and epigenetic differences in brain cells during aging in males and females. As a result, we hereby show that the presence of brain aging patterns in males and females is due to a complex of factors associated with the effects of sex chromosomes, which subsequently entails a change in signal cascades in somatic cells.

## 1. Introduction

The difference in life expectancy and the nature of aging between men and women is widely known. Women have a longer life expectancy, yet are more vulnerable to developing neurodegenerative diseases with the onset of menopause. However, the mechanisms underlying these differences are still not entirely understood [1]. The maintenance of viability is crucial for many cell types, especially for neurons, since mature postmitotic neurons must maintain their functional activity throughout the whole lifetime of a person. Previously it was assumed that cells in male and female organisms are biologically identical, and, therefore, to be cost-effective, all studies, including those that formed the basis of evidence-based medicine, were carried out predominantly on male animals [2]. However, later it became evident that many differences arose in men and women in response to various types of treatment, in addition to variations in humoral regulation, which are attributed to genetic, epigenetic, and hormonal sexual dimorphism [3,4].

**Figure 1:**
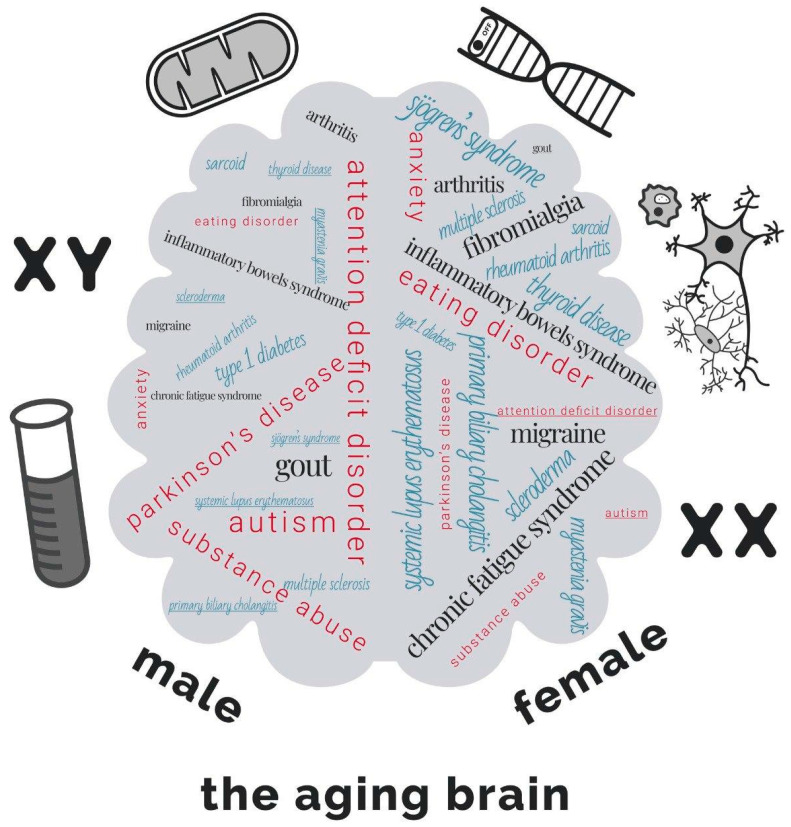
Sex differences in the incidence of diseases in men and women. The font size on a gray background correlates with the % of the incidence of the disease in a given sex [3]. Neurodegenerative or psychiatric diseases are shown in red font, autoimmune diseases are shown in blue font, and pain and social disabling disorders are shown in black font. An underlined font (e.g., male: Sjogren’s syndrome) corresponds to 5%, and the largest font (e.g., male: Myasthenia gravis) corresponds to 25%.

Indeed, differences in some aspects of cognitive functioning in older people and the vulnerability of women and men to various factors of dementia are reasonably well established [5,6]. For example, on average, women have a higher incidence of any dementia type [7]. Conversely, the prevalence of another progressive and age-related neurodegenerative disease, Parkinson’s disease (PD), is 1.5 times higher in men than in women [5] (Figure 1). Moreover, it was shown that the incidence and the mechanism of progression of Alzheimer’s disease (AD) in men and women are also attributed to sex-associated differences [8]. AD [9] and major depressive disorder [10] determined genetic correlates associated with sex. It is already known that women are more prone to exhibiting mood disorders such as depression and anxiety. At the same time, men are more susceptible to the dopamine system deficiency associated with PD, attention deficit hyperactivity disorder, and autism [11]. However, for neurological disorders, biological sex is still rarely taken into account in the decision of treatment [12]. Even though the differences in the prevalence of major chronic diseases between men and women are well established [3] (Figure 1), the molecular mechanisms underlying these differences currently require a more profound understanding.

The purpose of this review is to outline genetic and epigenetic factors forming sexual dimorphism of the brain during aging.

## 2. Genetic Effects of Sex Chromosomes in the Brain

The X and Y chromosomes exert direct genetic effects on the molecular physiology of the cell via their genetic products. During evolution, the functional role of the sex chromosomes did not differ from the autosomal before the appearance of features that determine sexual dimorphism [13]. Since the inheritance of sex chromosome genes, in particular, the Y chromosome in humans does not allow for genomic exchange, in the process of evolution over millions of years many chromosome functions have been lost. Sex chromosome genes are enriched in gene expression regulators that are sensitive to various substances’ concentrations [14].

The genes located on the sex chromosomes exert their effects not only on the sex cells but also on the physiology of autosomal cells [15] (Table 1). We describe that some of them have direct correlations with various brain functions. The genes located on the sex chromosomes that affect the central nervous system can be divided into three groups: (1) influencing through excitatory and inhibitory synapses; (2) influencing through the metabolism of steroids, including neurosteroids; (3) influencing cognitive processes, with an unclear mechanism of action. 

### 2.1. X-Linked Genes

The gene expression of the Duchenne and Becker muscular dystrophy gene (DMD gene), which is located on the X chromosome, has a direct effect on synaptic contacts. This gene encodes an actin-binding cytoskeletal protein involved in the assembly and maintenance of a set of GABAergic synapses [16]. The X-linked glycoprotein, synaptophysin [17], is located in the presynaptic vesicles. Some authors [18] suggest that the decrease of synaptophysin may reflect the number of neurons differentially affected by the neurofibrillary tangles pathology in dementia. The HSD17B10 gene mapped on the X chromosome has a triple effect and affects the cognitive functions of the brain through a change in the vulnerability of synaptic mitochondria to estrogen. HSD10 is a multifunctional mitochondrial enzyme that plays a significant role in the metabolism of neuroactive steroids and isoleucine. In the human brain, HSD10 catalyzes the inactivation of 17b-estradiol. Extremely high levels of HSD10 in synaptic mitochondria may lead to a state of local estrogen deficiency in the synapses, which makes them more vulnerable to stroke. This side effect can increase the risk of AD and impair learning and hippocampus-dependent memory [19]. A silent mutation (R192R) and three missense mutations (R130C, L122V, and N247S) in HSD17B10 cause X-linked mental retardation, choreoathetosis, and abnormal behavior (MRXS10) and hydroxyacyl-CoA dehydrogenase II deficiency, respectively. The latter condition appears to be a multifactorial disease due to the disruption of several metabolic pathways resulting from HSD10 deficiency [19]. There are other products of sex chromosomes’ genes that affect steroid metabolism. In particular, the STS gene expressing steroid sulfatase [20] plays a key role in regulating the formation of biologically active steroids [21], it is also associated with attention deficit disorder [22] and aggressive behavior [23]. As is known, sulfated and unsulfated steroids are modulators of GABA type A receptors [24] and NMDA receptors [25]. There are other genes of sex chromosomes that affect the cognitive functions of the brain, but their mechanisms of action are not yet well understood. For example, the PTCHD1 gene of the X chromosome [26], which is expressed in GABAergic and glutamatergic neurons in the thalamic reticular nucleus, is associated with sleep, sensorimotor processing, and attention [27]. Various pathologies were associated with altered expression of the listed sex chromosomes’ genes. For example, mutations in the PTCHD1 gene are considered to be one of the causes of mental retardation [28], since various disorders lead to nonsyndromic X-linked intellectual disability and/or autism spectrum disorder in males [29]. Many patients with Duchenne and Becker muscular dystrophy [30] have cognitive impairments, learning disabilities, and an increased incidence of neuropsychiatric disorders [31]. Another gene of the X chromosome, CASK, [32] is associated with mental retardation and microcephaly with hypoplasia of the brain in girls. CASK mutations in boys can cause epileptic encephalopathies, yet the mechanism of seizures remains unknown. Some missense CASK mutations in boys are milder and are usually found in cases of X-linked mental retardation in normocephalic boys [33]. Moreover, the non-coding transcript Xist is known to be important for the inactivation of the X chromosome in women [34], and the development of the central nervous system [35]. Pbdc1, Mid1, and Ddx2x are three genes known to avoid inactivation on the X chromosome in mice [36].

### 2.2. Y-Linked Genes

Y-linked genes mainly have regulatory significance as DNA-binding transcription factors that change the expression of other genes in autosomal cells, thereby affecting multiple molecular pathways. The most studied Y chromosome gene is the SRY gene [37]. An ontogenetic study of Sry expression in the brain of male mice shows that the circular, presumably untranslatable form, is expressed in 11–19 embryonic days [38]. After birth, linear and translatable forms of Sry are identified in the diencephalon, midbrain, and cerebral cortex. The transition from cyclic to linear transcripts is apparently due to the switching of promoter activation [39]. 

The Sry protein itself is increasingly observed in the substantia nigra of the midbrain and medial mastoid bodies of the hypothalamus in males, with weak levels of Sry expression throughout the cortex [40], and is localized exclusively in tyrosine-hydrolase-positive neurons with the distribution both in the cytoplasm and the nucleus of cells. The number of tyrosine-hydrolase-immunoreactive neurons decreases by 53% with age [41]. Thus, the number of SRY-positive neurons will also decrease with age, which may lead to a shift in the molecular pathways of the brain during aging in males.

Perhaps the largest genetic source of sexual dimorphism comes from the Y chromosome’s SRY gene, which controls testis development in males [3]. The rise and fall of testosterone are of paramount importance in programming sex differences in physiology and susceptibility to diseases that manifest in adulthood. The contribution of sex chromosomes and/or sex-specific gene expression was demonstrated by an elegant experiment [42]. In dissociated cultures derived from rat embryos at the time of formation of the sex gonads, when the effect of gonadal steroid hormones is negligible, differences were found between dopaminergic neurons in females and males. In particular, more dopaminergic tyrosine hydroxylase immunoreactive (TH-ir) midbrain cells were found in female cultures, and their mesencephalic and diencephalic neurons also produced more dopamine compared to males. However, male cultures contained larger dopaminergic neurons. The impact of estradiol and testosterone did not alter observed dynamics [42]. Sry encodes a transcription factor that activates the Sox9, Cbln4, Pod1, and Nt3 genes. The functions of Sry include the suppression of genes that form antibodies to the testes, the curvature of the DNA double helix, the role of the cofactor in gene silencing complexes, and the splicing factor for pre-mRNA; also the Sry transcript can function as a regulator for non-coding RNA (ncRNA) [39]. As is known, not only are ncRNAs involved in the spatial and temporal control of mRNA translation, which is necessary for functionally separated neurons [43], but they are also associated with brain development, neuronal differentiation, and complex functions such as learning and memory [44]. The role of genetic effects is confirmed by numerous failures in the work of the dopaminergic systems of the midbrain, characterized by significant sex differences in their prevalence and/or nature, which underlie many neurological and mental disorders, including schizophrenia, attention-deficit/hyperactivity disorder, autism, autism spectrum disorders, substance abuse, anxiety, and depression [12,45]. Experiments [46] with genetic manipulation of SRY have demonstrated the independence of lifespan from the development of the gonads, but with a dependence on the genotype, in particular, an increase in lifespan with a combination of XX chromosomes [46], regardless of the biological sex, in particular, in AD [47]. However, it is unclear how the presence of a Y chromosome reduces, or a second silent X chromosome increases, overall lifespan in mice.

### 2.3. Paralogous Genes of Sex Chromosomes

Even though SRY is distinguished among the main genes of sex chromosomes, there are other homologous genes contributing to the formation of sexual dimorphism, for example, genes for ubiquitin-specific proteases (Usp9x and Usp9y) originating from the X and Y chromosomes, respectively [48]. A study of sex differences in mice with the expression of Usp9x showed that Usp9x is found in the brains of embryos, newborns, and adult females, while the homologous gene from the Y chromosome (Usp9y) is expressed in newborn males, but less so in adults. At the same time, in adult females, Usp9x is specifically expressed in the neocortex, hippocampal subregions, and cerebellar Purkinje cells [49], where it participates in various processes, including synaptic development and plasticity, as well as self-renewal, differentiation, and migration of neural predecessors. Changes in the expression of these genes in autism have been studied quite recently [50].

There are several genes located on both the X and Y chromosomes. However, they also have their differences. Thus, the UTY [51] gene has differences in the intensity of expression between paralogs X and Y in the brain, which can lead to sex differences in brain functioning. Utx is expressed mainly in the amygdala, and Uty is expressed in the paraventricular nucleus of the hypothalamus [52]. 

A remarkable example of the Y chromosome’s role in brain development is two pairs of homologous X-Y genes encoding the synaptic cell adhesion molecules PCDH11X/Y and NLGN4X/Y, protocadherins and neuroligins, respectively [53]. Both genes regulate the contacts of pre- and postsynaptic sites through homo- or heterophilic interactions of their extracellular domains, mediate trans-synaptic signaling cascades, and regulate numerous aspects of synapse development, function, and plasticity that are necessary for neurotransmission. Thus, various neuroligin isoforms (NLGN) bind to presynaptic neurexins. NLGN 1-3 are highly conserved in mammals, and extensive studies in mouse models show that NLGN1 localizes to excitatory synapses, NLGN2 is in the inhibitory synapses, and NLGN3 can be found in both [54]. NLGN4 has a trafficking deficit that hinders its ability to induce synapses, due to its inability to move to the surface. The proof of the causal relationship of NLGN4X with Alzheimer’s and mental abilities is that the variant of the NLGN4X mutation can be present in males in the same pedigree for many generations, which is due to the inability of NLGN4Y to compensate for any deficiency in NLGN4X [55].

Various diseases can serve as proof of the connection of sex chromosomes with the physiology of the brain. There are paralogs differently involved in neurogenesis. The RPS4Y gene is located on the Y chromosome and encodes a universally expressed ribosomal protein gene. Interestingly, the expression of this gene increases during neural differentiation. XY males express two genes encoding RPS4 (RPS4Y and RPS4X), whereas XX females express only one copy of RPS4X due to X inactivation; thus, this indicates that the levels of the RPS4 protein in men compared to women are initially higher. This unevenness of distribution is associated with the development of autism spectrum disorders [56]. Another DDX3X gene has a paralog of the Y chromosome, DDX3Y, and the protein products of these two genes are more than 90% identical and mainly disturbances in their work are associated with sexual disorders [57]. Moreover, DDX3Y is assumed to be one of the candidates for physiological changes in the development of PD [58]. Moreover, one of the variants of the TBL1Y gene is considered to be a potential cause of hereditary hearing loss [59].

**Table 1:** Function and localization of sex chromosome genes that are essential for brain function.

Gene	Localization	Function	References
DMD	Xp21.2-p21.1	Encodes an actin-binding cytoskeletal protein. Distal DMD mutations are linked to cognitive impairment. The risk and severity of cognitive disability are associated with a cumulative loss of distal DMD.	[16,60,61]
SYP	Xp11.22-p11.23	SYP is an integral membrane protein of synaptic vesicles.	[62,63]
HSD17B10	Xp11.2	Affects the cognitive functions of the brain through a change in the vulnerability of synaptic mitochondria to estrogen.	[19,64]
STS	Xp22.31	A key role in regulating the formation of biologically active steroids, it is also associated with attention deficit disorder and aggressive behavior.	[21,22,23,65]
PTCHD1	Xp22.11	PTCHD1 is associated with sleep, sensorimotor processing, and attention. PTCHD1 is predicted to be a transmembrane protein that encodes the 12 transmembrane helices that form two modules. A distinct pattern of membrane localization within dendritic spines. In addition, a portion of the intracellular C-terminal tail encoded appears to be essential for dendritic and synaptic targeting.	[26,27,66]
CASK	Xp11.4	A role in a wide variety of cellular functions including transcription regulation, insulin signaling, and secretion. MICPCH is also considered a neurodevelopmental disorder that occurs due to heterozygous mutations in gene CASK in girls. Some missense CASK mutations in boys are milder and are usually found in cases of X-linked mental retardation in normocephalic boys.	[33,67,68,69]
SRY	Yp11.2 ^1^	The Sry transcript can function as a regulator for non-coding RNA (ncRNA). As is known, not only are ncRNAs involved in the spatial and temporal control of mRNA translation, which is necessary for functionally separated neurons, but they are also associated with brain development, neuronal differentiation, and complex functions such as learning and memory.	[39,43,44]
Usp9x/y	Xp11.4/Yq11.221 ^1^	Usp9x encodes a ubiquitin protease implicated in synaptic development, to be significantly higher in adult female mouse brains than in male brains.	[70]
UTX/Y	Xp11.3/Yq11.221 ^1^	Utx is involved in regulating HOX genes. Utx was expressed mainly in the amygdala, and Uty was expressed in the paraventricular nucleus of the hypothalamus.	[52,71]
PCDH11X/Y	Xq21.3/Yp11.2 ^1^	The protein plays a fundamental role in cell–cell recognition essential for the segmental development and function of the central nervous system.	[72]
NLGN4X/Y and RPS4X/Y	Xp22.3/Yq11.2 (NLGN4X/Y) and Xq21.3/Yp11.2 (PCDH11X/Y)	NLGN4X/Y genes encoding the synaptic cell adhesion molecules neuroligins. NLGN4 has a trafficking deficit that hinders its ability to induce synapses, due to its inability to move to the surface.	[53,55,73]
RPS4X/Y	Xq13.1/p11.31	The RPS4 gene codifies for ribosomal protein S4.	[74,75]
DDX3X/Y	Xp11.3–11.23/AZFa region on the Y-chr	DDX3X enhances transcription by interacting with transcription factors. DDX3Y is expressed more broadly in tissues across the human body. DDX3Y is assumed to be one of the candidates for physiological changes in the development of PD.	[57,58]
TBL1X/Y	Xp22.31-p22.2/Yp11.2 ^1^	One of the variants of the TBL1Y gene is considered to be a potential cause of hereditary hearing loss.	[59]

^1^ Gene localization from the National Library of Medicine (NCBI).

## 3. Expression of Non-Sex Chromosome Genes in the Brain

Over the past few years, many genes associated with the lifespan of mice have been discovered, and sex-specific molecular pathways have been identified [76]. Many of the genes associated with longevity are related to eight biological processes: cytoskeleton-dependent intracellular transport, metabolic processes associated with tRNA, cell cycle, cell morphogenesis, protein folding, cell division, the cellular amino acid metabolic process, and ribosome biogenesis. The mentioned set is significantly enriched in genes with binding sites for transcription factors HSF, ELK1, EFC (RFX1), USF1, and USF2 [77]. It was found [78] that in men, as in women, the expression of genes related to different types of synapses, calcium signals, and long-term potentiation does not correlate with age, whereas expression of genes associated with the extracellular matrix, cytoskeleton, and Hippo- and PI3K-Akt signaling pathways correlate.

**Figure 2:**
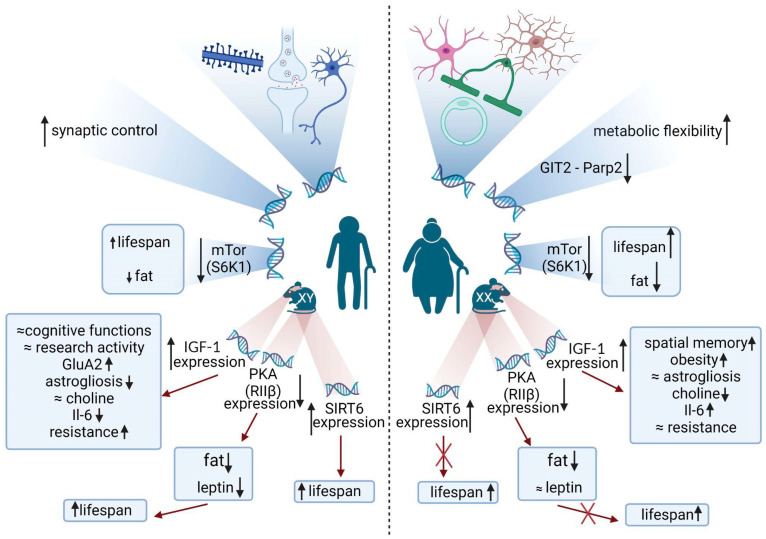
Sex-dependent molecular pathways associated with brain aging. During aging in males, genes associated with synapses, neurons, and dendrites are changed; however, in females, there are transcriptional changes in genes linked with microglia and astrocytes. Moreover, GIT2 gene expression changes in young age switch hypothalamus metabolic pathways that make females more resistant to metabolic changes in old age. Decreased activity through the mTor signaling pathway allows increasing lifespan more among females than males. In comparison to females in males, it is better to increase the male lifespan by decreasing the expression of RIIβ and increasing SIRT6 gene expression. However, some changes that are effective for prolongation of the lifespan in males are ineffective for females, for example, increased expression of the IGF-1 gene in females causes obesity, elevated IL-6 levels, and constant astrogliosis and resistance, whereas in males the same changes in IGF-1 gene expression cause an increase in GluA2 receptors and a decrease of astrogliosis and IL-6 levels with a constant value of choline. Created with BioRender.com.

The result of the above-mentioned study partially correlates with another [8] that evaluates the transcriptome of CNS cells and determined sex differences. Thus, in men, as they age, the activity of genes associated with neurons (25.28%), synapses (18.013%), and the development of dendrites (16.076%), macrophage genes (9.11%) changes more, whereas in women an inflammatory profile was spotted, characterized by modulation of genes belonging to astrocytes (13.741%), endothelial cells (22.474%), and microglia (14.828%) [8]. Changes in gene expression were three times more pronounced in males compared to females [71] (Figure 2).

Next, some genes are considered, the expression of which is differentiated depending on sex.

### 3.1. Changes in the Expression of Genes Associated with the Signaling Pathways of Insulin and Insulin-like Growth Factor-I (IGF-I), Collectively Called IIS (Insulin/IGF Signaling)

**Figure 3:**
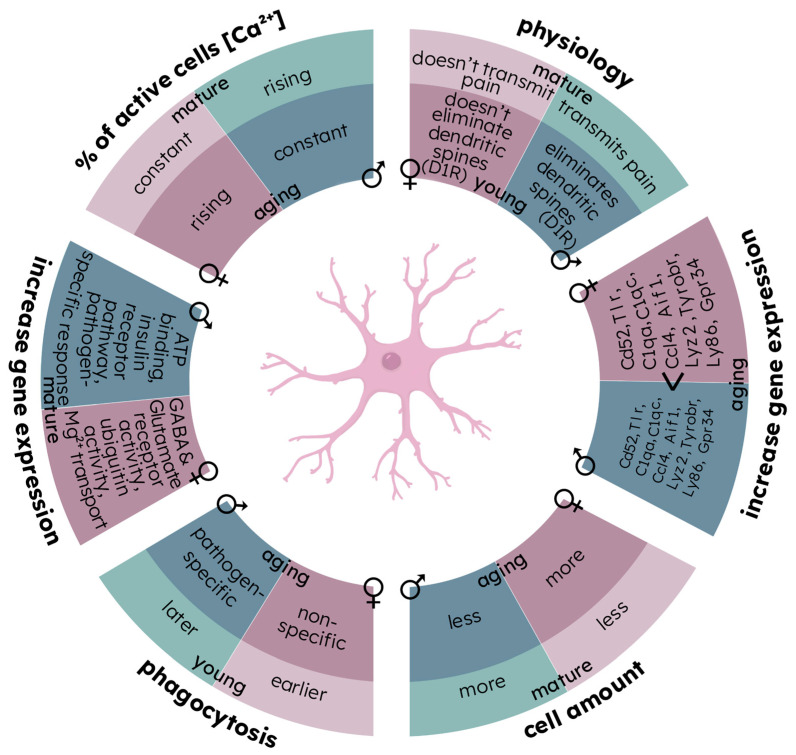
Sex-dependent differences in structure and function of microglia during normal development. During age, phagocytic activity changes: in females, phagocytic activity appears earlier than in males and unspecific phagocytosis increases with age significantly. However, in males, only pathogen-associated phagocytosis increased with age. At young ages among males, microglial cells participate in the elimination of dendritic spines containing D1 receptors, and in adult age in nociceptive transmission in the spinal cord, whereas in females microglia do not participate in such processes. Metabolic calcium activity of microglia is also different among males and females. In adult males there is an increase in the number of active cells; however, the total number of cells remains constant with age. On the other hand, among females, the ratio of active microglial cells remains constant up to old age, it is increased at this period of development. In female microglia, there are gene expression changes associated with ubiquitination, ionic magnesium transport, and GABAergic and glutamate activity in comparison to males. However, in males, the level of expression of genes of insulin receptors and pathogen-specific response is much higher in comparison to females.

Interestingly, short-term administration of IGF-1 improves neurogenesis and working memory in older males, but gene therapy is effective in older females, which emphasizes the importance of sex differences in brain aging. Increased expression of IGF-1 in the brain causes a different response in females and males during aging in such processes as obesity, spatial memory, endurance, astrogliosis, choline, and IL-6 concentration (Figure 3) [79]. Recent studies have determined the effect of sex on age-associated depressive symptoms, such as weakness, decreased muscle strength, and motor activity, as well as the role of intramuscular IGF-1 gene therapy in these processes [80]. A slight decrease in IGF-1 was used to increase the lifespan of dwarf mice; however, the effect may be due to a decrease in the transmission of growth hormone signals. Moreover, it was shown that increased insulin sensitivity after PTP1B (a negative regulator of the insulin signaling cascade) disabling and thus, attenuation of dephosphorylation of key intermediates in the insulin signaling cascade shortens life expectancy [81].

### 3.2. Changes in the Expression of Genes Responsible for the mTOR Signaling Pathway That Activate the Downstream Effector Kinase 1 of Ribosomal Protein S6 (S6K1)

Inhibitors of mTOR (target of rapamycin in mammals) or deletion of genes encoding components of its signaling pathway, including ribosomal protein kinase S6 (S6K1), increase lifespan [82]; however, in many studies, the effects were stronger in females [83,84]. This pathway is considered to be implicated in changing the volume of adipose tissue. Many researchers are inclined to believe that adipose tissues perform endocrine and immunological functions [85]. Obesity is known to lead to inflammation and, as a result, to the development of neurodegeneration [86]. In a recent study of insulin resistance, a difference was found between young females and males in the amount of activated S6K1 in several organs, but this difference disappeared in adulthood [87]. This phenomenon in the brain during aging has not yet been studied. In general, reduced/absent S6K1 activity leads to fat loss and increased longevity, which is not always sex-associated [88].

### 3.3. Changes in the Expression of Regulatory Genes

cAMP-dependent mammalian protein kinase A (PKA) consists of two regulatory and two catalytic subunits. Mice have four regulatory and two catalytic isoforms, each of which is encoded by a separate gene. RIIβ-null (RIIβ^-/-^) mice are leaner compared to their wild-type littermates, they have increased metabolic activity at rest, modified body temperature, a different concentration of uncoupling protein 1 (UCP1), and lipid hydrolysis. The correlation of the activity of this gene with life expectancy has been proven, in particular, a violation of PKA RIIβ in mice increases life expectancy and protects against age-related obesity, weight loss at the end of life, heart hypertrophy, frequency and severity of age-related pathology and insulin resistance in males [89]. Whether this relates to brain function or the cause is a different distribution of fat is yet to be understood [90].

### 3.4. Changes in the Expression of Sirtuins (SIRT6)

SIRT6 is considered to be an epigenetic regulator of great importance for the brain since proteins capable of maintaining neuronal genomic and epigenomic integrity throughout life are required there [91]. SIRT6 is reported to function as an NAD+-dependent H3K9 deacetylase. H3K9 modulates telomeric chromatin to ensure efficient telomere replication and prevent the accumulation of structural abnormalities in telomeres [92]. SIRT6 is also highly expressed in the CNS and its expression is regulated by nutrient availability, showing low levels in the hypothalamus in obesity [93]. 

Physical training increases the level of SIRT1 mRNA and the amount of mtDNA, which indicates an increase in mitogenesis in most areas of the brain with potential cognitive value [94]. Sirtuins appear to be involved in the lifespan-modulating effect of IIS [95]; the expression of SIRT3-SIRT7 changes in the aging brain depending on the brain region [96]. It was shown that single-nucleotide polymorphisms in the SIRT3, SIRT5, and SIRT6 genes correlate with human lifespan. At least one of the sirtuins was demonstrated to have sexual dimorphism. Thus, males, but not females of transgenic mice with overexpression of Sirt6 have a significantly longer lifespan than wild-type mice [97].

### 3.5. Heterozygosity of GIT2

Using bioinformatics approaches, the authors of [98] concluded that women at the hypothalamic level have an innate “program” of aging resistance with differential dependence on the aging regulator GIT2. Thus, higher life expectancy in women is associated with the ability of GIT 2 to control the flexibility of energy metabolism. On the contrary, in men, synaptic functions appear to be prioritized at the expense of metabolic flexibility. GIT 2 forms a dynamic complex with Parp 2 (poly(ADP-ribose)polymerase-2) in response to DNA damage [99]; therefore, it is not surprising that the decrease in Parp 2 observed in women [98] is associated with increased sensitivity to DNA-damaging agents in mice and humans. The work of these genes may cause, as a protective mechanism, the switching of energy metabolism from primary oxidative phosphorylation to beta-oxidation of fatty acids, which increases life expectancy in women [100].

## 4. Microglia as the Main Cellular Source of Sexual Dimorphism in the Brain

Microglial cells are macrophages of the central nervous system, also involved in the formation of synapses and the suppression of pathogens by the release of cytotoxic substances. These cells are now considered to be crucial in determining the difference in brain functioning between men and women [101,102]. In the physiology and morphology of microglia, many differences are found between men and women, including those associated with aging, as well as with the onset and development of neurodegeneration [103]. Sexual dimorphism is manifested in microglia with the following features: the number of cells during aging [104], participation in pain transmission [105], elimination of dopaminergic dendritic spines in adolescence [106], and phagocytic activity (at the beginning of development [107], and during aging [108]). Moreover, in comparison to microglia in males, higher levels of mRNA for TNFα, IL-1β, IL-6, and IL-10 were found in females [109].

The quantitative ratio of microglial cells differs at all stages of ontogenesis [110], which can affect the difference in synaptic contacts and the response to stress, learning, etc. In the late stages of development, activation of genes encoding some inflammatory proteins is observed in females (CCL4, CCL20, and CD206), as well as IL-1β, TNF-α, and CXCL10, unlike males [111], possibly due to the quantitative superiority of microglial cells in females during aging. The difference in gene expression in hippocampal microglial cells is observed at different stages of ontogenesis [112]. Recent studies have also shown that male microglia appear to be already more reactive under physiological conditions, but also have a shorter lifespan [113]. In addition to differences in the expression of inflammatory protein genes, the expression of sex hormone receptors in microglia increases with age [114]. At the same time, the reaction to sex hormones in male and female animals is different [115].

Interestingly, with aging, Ca^2+^ signaling undergoes different changes in male and female microglia. In vivo studies by Brawek et al. revealed differences in Ca^2+^ signaling, indicating more rapid aging of female microglia [116], which is supported by other studies [117]. Sexual dimorphism of microglia also includes the difference in the electrophysiological properties of the membrane in mature microglia [118] (Figure 3).

### 4.1. Pathogen-Associated Inflammation

Aging is known to make the brain more susceptible to systemic inflammation caused by lipopolysaccharides (LPSs) [119]. When exposed to lipopolysaccharides (LPSs), the initial morphological characteristics of the developing microglia in males are significantly reduced compared to females [120].

In the study by Doyle et al., it was also demonstrated that LPS exposure induced greater microglial activation in the periaqueductal gray matter of female rats compared to males and it was accompanied by increased levels of IL-1β transcription [121]. Moreover, female microglia produced higher levels of IL-1β transcription and were more sensitive to LPS than male microglia [122].

It is known that during neuroinflammation, microglial cells such as macrophages are capable of modifying metabolic functions and switching from oxidative phosphorylation to glycolysis [123]. According to recent data, this metabolic shift occurs when neonatal mouse microglia are activated by stimuli such as LPS, Aβ, and IFNγ, leading to an increase in PFKFB3, a key glycolytic enzyme [124]. Microglia derived from aged mice also exhibit increased glycolysis and increased levels of PFKFB3. In addition, microglia activation, with a corresponding metabolic switch, is sex-dependent and is characterized by more profound changes in female cells compared to male APP/PS1 mice [124].

### 4.2. Traumatic Brain Injury (TBI) and Obesity

Obesity and diabetes are known to lead to an increased risk of dementia with aging [125]. In addition, the impact of protein quantity and quality on health and metabolic pathways, and the association between insulin resistance and obesity has been discussed [126]. In obesity, microglial cells are activated in various areas of the brain, first of all, in the hypothalamus and hippocampus, which further leads to an alteration of cognitive functions. A recent study demonstrated a high resistance of females receiving a high-fat diet to obesity by maintaining the level of Cx3Cl1-Cx3Cr1 (the chemokine fractalkine receptor C-X3-C motif 1) and its highly selective Cx3Cl1 ligand, known as the Cx3Cl1–Cx3Cr1 axis [127], while male mice show a decrease in ligand and receptor expression. Female mice with Cx3Cr1 knockout develop “male” accumulation and activation of microglia in the hypothalamus, which is accompanied by a noticeable increase in their predisposition to obesity caused by diet. Microglia may be associated with metabolic disorders [127] through the modulation of energy homeostasis because it demonstrates the expression of genes of glycolytic and oxidative metabolism. Interestingly, the effect of arachidonic acid on obese males showed an increased amount of Iba-1 microglia and mRNA levels for Iba1, TNF-α, IL6, and TLR4, which indicates activation of microglia [128]. Interestingly, no such changes were observed in females.

A more unfavorable outcome after TBI was found in male mice, while obese female mice showed the same amount of microglia activation before and after TBI. Moreover, compared with male mice with TBI and obesity, females were found to have lower microglial activity [129]. A possible explanation for this observation is the sex-specific resistance in females to diet-induced activation of microglia-mediated by the aforementioned Cx3Cl1–Cx3Cr1 axis. At the same time, deletion of the Cx3Cr1 fractalkine receptor in obese females led to rapid activation of microglia [127]. These results indicate that fractalkine has an inhibitory effect on the activation of microglia in obesity.

### 4.3. Proteinopathy

In the process of microglia sequencing in tauopathy [130], in addition to differential expression of sex chromosome genes such as Xist, Ddx3y, Uty, and Eif2s3y, it was found that microglia in males had a greater enrichment of genes involved in inflammation and phagocytosis, including Spp1, Ccl6, Lpl, Il1b, and Cst7, which is characteristic of the disease-associated development of microglia. However, another study claims [131] that there is no relationship between sex and age in tauopathy, but there are significant differences in the expression of microglia genes in amyloidosis. In general, women with AD have a greater tendency to have cognitive impairment and the development of the disease. Of the genetic differences found, the expression of 13 sex-related long non-coding RNAs should be noted [132], and the activation of expression of several microglial genes in AD [133].

Age and sex are known as major risk factors for developing Alzheimer’s disease (AD). A higher incidence of AD is observed in women [134], which is linked with neuroinflammation and inadequate activation of microglia [135]. Changes in genes that indicate microglial activation were discovered by Guillot-Sestier et al. in female mice of the APP/PS1 transgenic line. Their microglia were glycolytic, less phagocytic, and associated with increased amyloidosis, while male microglia were amoeboid [135]. Aging female microglia have a greater ability to phagocytose neuronal debris, but lose the ability to adapt their phagocytic activity to inflammatory conditions [5]. In addition, female mouse microglia show increased expression of an APOE-driven network of genes associated with aging, amyloidosis, and tau protein aggregation [136].

In a recent study, transcriptomic and proteomic analyses of microglia from five different brain regions of male and female C57BL/6J mice were performed. The expression of major histocompatibility class I and II genes was higher in the cortical microglia of males than females, suggesting a higher antigen-presenting ability [137].

Studies on the relationship between microglial activation, toll-like receptor 4 (TLR4), and novel tyrosine kinase Lck/Yes (LYN) signaling in a mouse model of AD show a correlation between microglial TLR4 and LYN colocalization and AD pathogenesis, greater in females than in males of 5XFAD mice [138]. Recent studies also show that there are sex-related differences in miRNAs expressed in microglia that lead to sex-specific changes in the transcriptome and tau pathology [139]. For example, 61 miRNAs are expressed differently in microglial cells of male and female mice [130].

In addition, the aforementioned tauopathy, amyloidosis, and aging share a common transcriptional signature that APOE creates in microglia. This fact indicates that the increased expression of many of these microglial transcripts may be associated with increased susceptibility to AD in women [140].

### 4.4. Hypoxia/Ischemia

It is known that more women die from ischemic stroke than men, which highlights the significant sexual dimorphism in the pathophysiological outcomes of ischemia [141]. Indeed, in a mice model of ischemia, young adult female mice and rats suffer less in comparison to males. Moreover, female microglia showed a neuroprotective phenotype during ischemic stroke [142]. However, mice with estrogen receptor α (ERα) knockout (postmenopausal brain model) showed dysfunctional activation of microglia and increased proinflammatory response after ischemic injury [115].

Complementary to this, in a mice model of acute ischemic injury by middle cerebral artery occlusion there was a strong activation of microglia with increased Iba1 immunoreactivity among males, and an enlarged surface of the brain affected by strokes in comparison to females [142]. It can be seen that such a phenomenon is due to different gene expressions of microglia among males and females. According to some authors’ data observations, there are sex-biased transcriptional differences in microglia; thus female microglia demonstrate a neuroprotective phenotype that persists after transfer into the male brain [143].

## 5. Sex-Dependent Neuroendocrine Aspects of Aging

Many authors state that the mechanisms of aging and longevity have a connection with gonad activity and neuroendocrine signals. In the brain, sex steroid receptors are mainly located in the limbic system, especially in the hippocampus. In this connection, changes associated with menopause/andropause can change the structure and function of the brain during aging [144]. Moreover, it has been suggested that the deterioration of the production of sex hormones contributes to the accelerated phenotype of aging [145]. 

At the same time, the nervous system itself is a source of various neuroactive steroids (neuronal estrogens and androgens), which are synthesized and metabolized by neurons and glial cells. However, the levels of neuroactive steroids and enzymes are different in the nervous system of males and females [146]. Different levels of expression of neurosteroids, as well as their receptors and enzymes, can serve as one of the reasons for the dimorphic pattern of aging in male and female brains.

**Figure 4:**
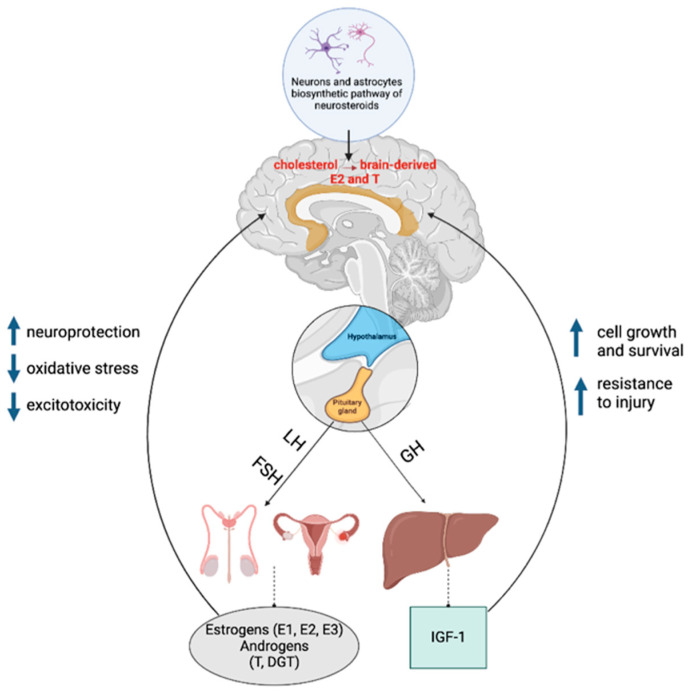
General scheme of production and effects of sex hormones and IGF-1 on the brain (LH—luteinizing hormone; FSH—follicle-stimulating hormone; GH—growth hormone; IGF-1—insulin-like growth factor 1; E1—estrone; E2—estradiol; E3—estriol; T—testosterone; DGT—dihydrotestosterone). Created with BioRender.com.

Thus, it is assumed that there are certain pathways and mechanisms underlying neuroendocrine dimorphism in the aging of the brain (Figure 4).

### 5.1. Estrogens (E)

Sex hormones, in particular E, have powerful antioxidant properties and play an important role not only in maintaining reproductive function but also in providing neuroprotective action [147]. It is well known that E levels positively correlate with the density of dendritic spines in rodents, as well as with the degree of axon germination [148]. That is why their decrease during aging also leads to mitochondrial dysfunction, neuroinflammation, and cognitive impairment in both sexes [149].

Estradiol (E2) is considered to be the most physiologically significant E since it has the highest affinity for intracellular estrogen receptors, ERα and ERβ. In addition to the classic estrogen receptors, E2 also has a high affinity for the membrane-bound G protein associated with the GPR30/GPER1 receptor [150]. 

In women after menopause, there is an increase in markers of inflammation such as IL-1, IL-6, and TNF-α. However, this increase is normalized after treatment with sex hormones [150], since membrane-bound ER can activate the signaling pathways PI3K/PLC, MAPK/ERK, and cAMP/PKA, which are associated with neuroprotection [148]. It is assumed that E2-induced neuroprotection may be caused by the involvement of innate immune molecules through genomic regulation of ERβ [151] and an increase in the Bcl-2 apoptosis regulator while protecting against excitotoxicity [148].

Depletion of endogenous sex steroids in female rodents by ovariectomy significantly increases the level of soluble Aβ in the brain, which leads to the development of symptoms of AD [152]. Cui J. et al. have shown that E can reduce the production of Aβ and the level of hyperphosphorylated tau protein by modulating kinases and phosphatases involved in tau phosphorylation [153]. 

ER can activate the production of brain neurotrophic factor (BDNF), which protects against ischemic damage in vitro and preserves cognitive function [148]. In addition, the introduction of E2 after ovariectomy promotes the development of the protective phenotype of microglia and restores the semaphorin content in the hippocampus [151]. E can also reduce the level of hyperphosphorylated tau protein by modulating kinases and phosphatases involved in tau phosphorylation, such as the GSK-3β, Wnt, or PKA pathways [153].

### 5.2. Sexual Dimorphism of the Dopaminergic System

Studies have shown that ERα is also a dopaminergic receptor. Mice with ER genetic depletion are more vulnerable to stroke-induced cell death in the PD model [153]. Perhaps due to differences in the distribution of ER in dopaminergic neural networks (for example, associated with the expression of the SRY gene in men), men are more susceptible to schizophrenia and the age of onset occurs earlier than in women [154]. With neurodegenerative disorders such as AD, men have a tendency to antisocial behavior and women have a tendency to depression and delirium [155].

Based on this, it is possible to assume a specific role of ER in the dopaminergic system, which in turn may indicate the existence of sexual dimorphism during the aging of this system in the brain in men and women.

### 5.3. Testosterone (T)

Like E, T is also produced by neurons and glial cells, and its metabolites affect the functioning of brain cells. In rodents and humans, T is converted to E2 in the brain by the aromatase enzyme p450, which ultimately acts on ER, through which androgens such as E exhibit their neuroprotective properties [156]. T is also reduced to dihydrotestosterone, which acts directly through androgen receptors (ARs) [157]. 

In male rats, it was shown that an age-dependent decrease in T accompanies a disruption of spatial memory, which, however, can be restored by the administration of exogenous T [158]. Significant for the direct effect of T on cognitive functions, AR is expressed in the neurons of the hippocampus and amygdala. In vitro, in primary cultures of hippocampal neurons treated with T, a decrease in Aβ secretion was demonstrated [148]. Moreover, in vivo experiments have shown that the introduction of exogenous T increases synaptic density in the inner molecular layer of the dentate gyrus of the hippocampus of 27-month-old male Balb/c mice, regardless of exercise or calorie restriction [159]. The administration of T also significantly increases the amount of gray matter in the frontal cortex. However, in high doses, T causes the loss of dopamine neurons under conditions of oxidative stress, which may be associated with an increased risk of PD in men [160].

### 5.4. The Interaction of Sex Steroids with the Growth Hormone (GH)/Insulin Growth Factor 1 (IGF-1) System during Aging

The role of the GH/IGF-1/insulin system in the regulation of life expectancy is a widely discussed topic in gerontology since this pathway plays an important role in the pathogenesis of several age-related diseases, including dementia and metabolic diseases [161]. GH and IGF-1 stimulate protein synthesis in neurons, glia, oligodendrocytes, and Schwann cells and promote neuronal survival by inhibiting apoptosis [160]. The main effect of GH is that it stimulates the secretion of insulin-like growth factor-1 (IGF-1) from the liver [1]. 

Sex steroids regulate the tissue-specific effects of GH and IGF-1. T enhances the individual anabolic actions of GH and IGF-1, whereas E2 can both counteract and enhance the transmission of GH/IGF-1 signals depending on the target tissue [162]. The interaction of E2 with ER in peripheral target tissues is further modulated by feedback with GH and IGF-1 [163]. Disruption of the GH signaling pathway increases insulin sensitivity, which is associated with both delayed aging and an increase in life expectancy [164]. 

In patients suffering from AD, the GH/IGF-1 axis is suppressed [160], and reproductively aging women usually have a lower level of expression of cerebral IGF-1 and are prone to the formation of Aβ. This fact suggests the presence of a mechanism of synergistic interaction between ER in the brain, the insulin receptor, and the IGF-1 receptor (IGF-1R) [165]. Androgens also stimulate the GH/IGF-1 axis in men [166], at the same time, there is little information in the literature about the effect of androgens on the GH/IGF-1 axis in women. In men, the concentration of T positively correlates with the regularity of GH secretion, while a decrease in the level of T as a result of the natural aging process leads to an increase in the level of visceral fat and the development of metabolic syndrome [163]. At the same time, the results of the study show that lower IGF-1 levels are associated with low physical performance in men to a greater extent than in women [167]. 

Thus, the efficiency of the GH/IGF-1 axis is related to both the level of T and E2 in plasma. For example, IGF-1 gene therapy has a positive effect on the depression-associated decrease in locomotor activity in physiologically old (19 months) male mice, but not in females [80]. This data highlight important sex differences in how IGF-1 action affects life expectancy and cognitive health.

### 5.5. The Influence of Sex Hormones on Epigenetic Processes

Sex-related gene expression differences defined by hormonal activation of neuronal steroid receptors [168] can be observed in aging too. As mentioned above, estrogens and androgens interact with their nuclear receptors (ER and AR). Subsequently, the binding of sex hormones with the same nuclear receptors attracts coactivators, corepressors, and epigenetic modifier enzymes to promoters of target genes [169]. Upon binding to its ligands, nuclear hormonal receptors have a high affinity to certain DNA sequences, called hormonal response elements (HREs) [170]. Hence, the presence of HREs in the promoter region of target genes, particularly elements responsible for estrogen or androgen, called EREs or AREs, respectively, can explain the direct influence of hormones on transcription [171]. 

Many immune cells such as B- and T-lymphocytes, dendritic cells, and macrophages express ER [172]. Therefore, sex hormones’ action on the epigenome of innate immune cells can cause inflammation in aging cellular populations [169]. For example, DNA methylation and gene expression changes were observed in peripheral monocytes during menopause [170]. 

In the study of Frick K.M. et al., it was shown that epigenetic processes such as histone acetylation and DNA methylation were needed to provide E2-mediated enhancement of new object recognition memory in young mice after ovariectomy [173]. After E2 infusion in the dorsal hippocampal area, significant acetylation of histone H3 was observed in the ERK-dependent signal pathway, which is essential for improved object recognition [173].

However, besides classical histones, their nonallelic variants also work as epigenetic regulators [174]. This can be seen in the work of Ramzan F. et al. that AR overexpression inhibits fear memory in male mice and increase expression of the H2A.Z variant, a new epigenetic memory regulator [175]. These authors also found that conditional inducible deletion of H2A.Z inhibits memory-enhancing effects in the case of androgen depletion, e.g., after gonadectomy or pharmacological inhibition of AR [175].

In summary, this evidence shows a hormonal environment that is formed by estrogens and androgens capable of changing age-dependent epigenetic patterns. Subsequently, generated sexual differences may result in cognitive and behavioral peculiarities and immunological transition to menopause and andropause related to higher levels of inflammatory cytokines such as IL6, which points to the potential role of epigenetic changes in inducing chronic neuroinflammation [170,176].

Indeed, for this assumption, there is evidence that E2 can regulate the immune response in the brain by binding with ER of astrocytes and microglial cells [177]. Moreover, its deficits can cause the age-biased microglia phenotype to transition to an active state by such epigenetic mechanisms as DNA methylation, post-translational histone modifications, and miR activity [178]. Furthermore, E2 can regulate transcription and translation, contributing to the formation and modulation of dendritic spines, thus altering learning and memory [179]. E2 presumably promotes a transcriptionally permissive chromatin state. However, menopause and aging can cause the formation of a transcriptionally repressive chromatin state that would decrease the expression of genes associated with synaptic plasticity. Briefly, it will cause memory loss and thus susceptibility to certain senescence-related pathologies.

## 6. Sex-Dependent Regulation of the Mitochondrial Genome of the Brain

The mitochondrial genome is one of the crucial genetic factors that determine the functional sexual dimorphism of the human brain [180]. Mitochondrial inheritance occurs through the maternal line, and, as a result, mtDNA mutations that have a negative impact on the functional state of the female body are selected, while those affecting the male body accumulate [181]. The accumulation of deleterious mutations in the mitochondrial genome of men, a phenomenon called “selective sieve” and “mother’s curse”, is considered the basis of sex-associated specific patterns of aging [182,183]. Thus, due to the different inheritance, it is assumed that women will have better control over the performance and maintenance of mitochondrial functions than men [184]. Indeed, using animal models it was shown that in the brain, skeletal muscle, cardiomyocytes, liver mitochondrial gene expression levels, protein content, and overall activity is higher in women than in men [185,186]. Moreover, total fat distribution was strongly related to impaired monocyte and muscle mitochondrial function, but only in males [187].

In addition, hormonal regulation plays an important role in the formation of mitochondrial sexual dimorphism in the brain. Local synthesis of neurosteroids occurs in the brain [188], and the initial stages of steroidogenesis are localized in mitochondria [189]. mtDNA contains estrogen-associated regulatory sites (ERE: estrogen response elements), which upon interaction with the estrogen receptor (ER) increases mtDNA gene expression and respiratory chain activity [190]. Estradiol is known as an important regulator of the functional state of mitochondria. The interaction with the ERβ receptor localized on mitochondria leads to changes in mtDNA expression, the activity of the antioxidant defense system, and the oxidative and Ca^2+^ capacities of mitochondria [191]. In addition, among the factors leading to the formation of a sex-associated pattern of aging, there is a higher level of damage caused by the action of ROS (reactive oxygen species), due to their increased formation and a decrease in the activity of the antioxidant system in males compared to females [192]. Since the sites of ROS generation in the mitochondrial respiratory chain (complexes I and III) are located close to mtDNA, the mitochondrial genome, compared to the nuclear genome, is more vulnerable to oxidative damage [193]. In this regard, it was shown that the interaction of estrogens with the ERα receptor has a protective effect, reducing the level of ROS formation and increasing the levels of mitochondrial cytochrome c and mRNA in the brain endothelium [194]. Allopregnanolone (3α,5α-tetrahydroprogesterone) also showed positive effects on ATP levels and mitochondrial respiration in neurons, which is relevant for females [195]. Unlike female sex hormones, the regulatory role of testosterone and other androgens in the formation of mitochondrial sexual dimorphism in the brain remains relatively poorly understood [196].

As we age, the decline in sex hormones takes a toll on both the male and female bodies. However, in women, this decrease is more pronounced [196], and, therefore, the decrease in estrogen levels, in particular estradiol, with age in women (especially after menopause) is considered a significant risk factor during aging, leading to mitochondrial dysfunction [197]. However, it is shown that in men, with age the overall resistance to stress factors decreases to the greatest extent, and this was associated with a decrease in the number of mtDNA copies observed in Drosophila melanogaster [184]. A distinctive feature of late aging in various parts of the brain is the suppression of mitochondrial metabolism genes, in particular, those involved in electron transport, oxidative phosphorylation, mitochondrial transport, and ATP synthesis, which is more pronounced in men [198,199]. The dysfunction of mitochondrial metabolism is associated with the development of age-related CNS pathologies. Concerning AD, PD, and mitochondrial pathologies, mitochondrial haplogroups for men and women associated with an increased or reduced risk of developing the disease have been identified [200,201]. Proteomic analysis of the brain of patients with AD and cerebrovascular diseases demonstrated a decrease in the level of proteins of complexes I, III, and IV of the respiratory chain in women and complex II in men [202].

Finally, it should be noted that the main mitochondrial proteome contains about 419 conserved proteins, and only 13 of them are encoded by mtDNA, including proteins of complexes I, III, IV, and V of the respiratory chain [203], and thus, the discussion of the contribution of mtDNA genetic variability to mitochondria function throughout the life of the organism continues [204]. Mutations of nDNA are important concerning the mitochondrial variability of the organism since they can be compensatory to the action of the “selective sieve” [181].

## 7. Sex-Dependent Epigenetic Regulation of the Brain during Aging

Epigenetic regulation suggests changes in gene expression without changes in DNA sequence. Epigenetic regulatory mechanisms include DNA methylation, histone modification, miRs, lncRNA, and X-chromosome inactivation. [205]. 

### 7.1. Role of Histone Modifications

Histones can be modified by the covalent binding of methyl groups, phosphorylation, acetylation, ubiquitylation, and sumoylation, and together, these mechanisms are responsible for the regulation of chromatin structure and transcription of genes [206]. Notably, Wang et al. (2018) showed that the level of histone acetylation is connected with aging processes [207].

Levels of histone acetylation are maintained by the balance between histone acetylase and histone deacetylases (HDAC) enzymes [208]. According to preclinical and pathoanatomical research, an increase in HDAC gene expression and accumulation of hypo-acetylated histones is linked with aging and neurodegenerative diseases [209]. In the study of Gilbert et al. (2019), using low-invasive (11C) Martinostat, a radioactive PET tracer for HDAC expression, a sex-specific accumulation of tracer (SUVR—standard uptake value ratio) was found in different brain regions, which increased with age. SUVR was higher in females compared to males in the white matter of the frontal medial cortex, amygdala, hippocampus, parahippocampal gyrus, and thalamus. SUVR was lower in old females compared to old males in cerebellar white matter [210].

Histone methylation is the process of linking methyl group to lysine or arginine amino acid residue. Here, in comparison to histone acetylation and phosphorylation, lysine residue can be mono-, di-, or tri-methylated, and arginine residue can be mono-, symmetrically, or asymmetrically methylated [211].

In the study of Linnerbauer et al. (2020), it was demonstrated that astrocytes of old females after ischemia have elevated H3K4-specific methyltransferase compared to middle-aged females and males [212]. Moreover, astrocytes of old females after ischemia work as a source of micro-RNA, and H3K4me3 histone peaks in the mir 17–92 clusters were enriched in old females in comparison to middle-aged females [213]. Moreover, astrocytes of old females showed a decreased level of H3K4me3 modified histones in the VEGF gene in comparison with middle-aged females and males [214].

### 7.2. Role of DNA Methylation

DNA methylation is the process of covalent binding methylene group to the fifth residue of cytosine nucleotide residue that can change the expression state of genes. In the work of Pellegrini et al., they studied how the global level of DNA methylation changes in different parts of the brain among females and males during aging—they found that males have higher levels of DNA methylation in the frontal cortex, temporal cortex, and cerebellum in comparison to females but all sexes undergo hypermethylation with age. GO analysis revealed only one ontology enriched in the frontal cortex (“homophilic cell adhesion via plasma membrane adhesion molecules”) [215].

Another study showed the presence of epigenetic acceleration according to different epigenetic clocks among elderly patients with/without cognitive decline in both sexes—skin and blood age acceleration was significantly associated with the interaction of sex and diagnosis. They found increased age acceleration in CI females relative to CN females, but no effect in males. Age acceleration was significantly higher in male samples [216].

### 7.3. Role of miRs

MicroRNAs (miRs) are small (22 nt) RNAs that can post-transcriptionally regulate protein expression by inhibiting mRNA translation or causing mRNA degradation [217].

In the work of Rani and coauthors, it was shown that after induction of sepsis, there was activation miRs that enhance neuroprotection and inhibit neuroinflammation in young and old males and females. However, both young and old females showed activation of estrogen receptor signaling-associated miRs that led to better recovery of females [218].

### 7.4. Role of lncRNA

Long non-coding RNAs (lncRNAs) are a subgroup of RNA longer than 200 nucleotides (nt) that have limited protein-coding potential; however, lncRNAs can be epigenetic and transcriptional regulators that serve as scaffolds for the assembly of chromatin- and gene-regulating complexes and can take part in directing those complexes to specific loci in the genome [219]. 

Liu and coauthors (Liu et al., 2017) performed comprehensive RNA-seq and sex and age-biased analysis of lncRNA in rhesus macaque brain; they found a few sex and age-biased lncRNAs. However expression of lncRNA in sex-temporal modules was specific to both sex and age but was less associated with specific structures, among them AC027613.1, NONGGOT004660.1, and AC132825.2 were abundant in the macaque brain. Further analyses of RNA-seq, qPCR, and ISH data revealed a high correlation of the AC027613.1, NONGGOT004660.1, and AC132825.2 with sex and age specificities [220].

In the final analysis, it should be noted that due to presence of estrogen signaling in females have additional protective mechanisms that can affect different epigenetic mechanisms too, e.g., in the case of sepsis or with age or cognitive decline. As for epigenetic factors that can play a role in neurogenesis and brain development (e.g., lncRNA, histone modifications), these data show that female and male brains have different patterns of development and senescence.

## 8. Conclusions

The formation of a specific pattern of brain aging in men and women is highly influenced by both genetic and epigenetic factors. A considerable impact in that formation is exerted by both sex chromosome genes and changes in the activity of genes associated with crucial signaling pathways, such as insulin/IGF-1, mTOR signaling, and changes in sirtuin expression. In addition, another important source of brain sexual dimorphism is microglia, the quantitative ratio of which begins to differ in the male and female organisms even during development. Further, in the brain of females with age, an increase in the expression of inflammatory protein genes is observed. At the same time, they are distinguished by greater resistance to hypoxia/ischemia and traumatic brain injury, which may be associated with an increase in the expression of sex hormone receptors on microglial cells.

In addition, brain cells not only contain receptors for sex hormones but are themselves capable of synthesizing sex steroids de novo. Neuroendocrine differences in aging associated with andropause and menopause may have different implications. Thus, for example, men become more prone to a decrease in the number of dopaminergic neurons, and women have an increased tendency for the formation of Aβ.

Moreover, the initial stages of steroidogenesis are localized in mitochondria, and the activity of the respiratory chain itself is associated with the estrogen receptor. This may be related to the fact that the aging of the antioxidant system is more pronounced in the male body. Moreover, in neurodegenerative and cerebrovascular diseases, the dimorphism in functional changes of the respiratory chain complexes has been established in men and women.

It should be noted that due to the presence of estrogen signaling, females have additional protective mechanisms that can include different epigenetic mechanisms (e.g., miRs associated with activation Erα or anti-inflammatory genes) and epigenetic factors that can play a role in neurogenesis and brain development (e.g., lncRNA, histone modifications). These data show that some areas of female and male brains have different patterns of development and aging.

However, despite the considerable evidence for brain sexual dimorphism during aging, many mechanisms and physiological interactions remain hypothetical and require further study.
